# Analysis of Short-Term Clinical and Functional Outcomes in Patients Undergoing Total Knee Arthroplasty with Kinematic Alignment Technique

**DOI:** 10.3390/jcm12123978

**Published:** 2023-06-11

**Authors:** Riccardo Giorgino, Alessandra Nannini, Edoardo Scuttari, Alessandro Nuara, Ricardo Ciliberto, Corrado Sosio, Paolo Sirtori, Giuseppe M. Peretti, Laura Mangiavini

**Affiliations:** 1 Residency Program in Orthopaedics and Traumatology, University of Milan, 20141 Milan, Italy; 2 IRCCS Istituto Ortopedico Galeazzi, 20161 Milan, Italy; rciliberto@gmail.com (R.C.); sosiocor@gmail.com (C.S.); paosirto@libero.it (P.S.); giuseppe.peretti@unimi.it (G.M.P.); laura.mangiavini@unimi.it (L.M.); 3 Faculty of Medicine and Surgery, University of Milan, 20122 Milan, Italy; 4 Dipartimento di Scienze Biomediche per la Salute, Università degli Studi di Milano, 20122 Milan, Italy

**Keywords:** total knee arthroplasty, total knee replacement, kinematic alignment, knee osteoarthritis, clinical outcomes

## Abstract

Background: Surgery remains the best option for more advanced stages of knee osteoarthritis (OA). Kinematic alignment (KA) is an innovative surgical technique that aims to co-align the rotational axes of the femoral, tibial, and patella components with the three kinematic axes of the knee. This study aims to evaluate and analyze short-term clinical, psychological, and functional outcomes in patients undergoing total knee replacement with the KA technique. Methods: Twelve patients who underwent total knee replacement surgery with kinematic alignment from May 2022 until July 2022 were prospectively followed and interviewed. Before surgery, the day after surgery, and postoperative day 14, the following tests were evaluated: VAS, SF-12 PS, SF-12 MS, KSS, KSS-F, PHQ-9, and KOOS-PS. Results: The mean BMI value of 30.4 (±3.4) Kg/m^2^, mean age of 71.8 (±7.2) years. All the scores on the various tests administered consistently showed statistically significant improvement, not only immediately after surgery but also comparing the first to the fourteenth postoperative day. Conclusion: Kinematic alignment technique as a surgical treatment for KO allows the patient a fast postoperative recovery and good clinical, psychological, and functional results in a short time. Further studies are needed with a larger sample size, and prospective randomized studies are essential to compare these results with mechanical alignment.

## 1. Introduction

Osteoarthritis (OA) is the most common degenerative joint disease worldwide and the leading cause of disability in the adult population, characterized by cartilage degradation, subchondral damage, and bone remodeling [1,2,3]. Knee OA is the most prevalent site, accounting for most of the disease burden, and is commonly caused by prolonged survival, increasing rates of obesity, and joint trauma [4,5]. Management involves a comprehensive history, physical examination, and radiological investigation, with treatment options that can be divided into non-surgical and surgical interventions [6,7,8]. Surgical options include joint preserving and joint-replacing procedures, with total knee arthroplasty (TKA) being the most common procedure [9,10]. The goal of TKA is to reduce pain perception, restore knee function and mobility, and it is predicted that by 2030; 3.8 million people will undergo TKA every year [11,12]. The classical mechanical alignment technique, which has been used for more than 30 years, is designed to create a hip-knee-ankle (HKA) angle minor than 3° of the neutral mechanical axes [13]. However, it is evident that the mechanical alignment technique does not restore the knee’s physiological characteristics and simulates the knee joint’s motion pattern only on a two-dimensional plane, leading to possible knee tightness and stiffness [14,15,16,17,18,19,20,21]. Based on this theory, kinematic alignment (KA)-TKA was proposed in 2006 [9]. KA-TKA aims to co-align the rotational axes of the femoral, tibial, and patella components with the three kinematic axes of the knee, restoring the pre-arthritic or native constitutional alignment [17,18]. In KA, the orientation and height of the bone cuts are conducted by referencing the articular surfaces, compensating for cartilage and bone loss, and considering the thickness of the implants. The femoral and tibial components are positioned coincident with the native tibial–femoral joint lines, restoring the native laxities, tibial compartment forces, Q-angle, and hip-knee-ankle (HKA) angle [22]. The restoration of the native left-to-right symmetry of the HKA angle, distal lateral femoral angle, and proximal medial tibial angle are achieved by compensating for the 2 mm of cartilage worn at 0 and 90 degrees and adjusting the tibial resection until the varus–valgus laxity with trial components is negligible in full extension [23,24]. KA-TKA leads to a postoperative joint line orientation such as the native one and horizontal to the floor in a single-leg stance, resulting in a lower knee adduction moment than mechanically MA-TKA [25].

In recent years, several randomized controlled trials have compared patients with KA-TKA and those with MA-TKA. Patients with KA have reported better function in the Knee Society Score (KSS) and in the Oxford Knee Score (OKS), significantly better pain relief, and a more normal feeling knee [26,27,28]. In the recently published meta-analysis by Xu et al. [29], patients with KA-TKA had a difference of seven points in the knee function score, a better range of motion of 4°, and a better combined KSS with a mean follow-up of 12 months. Although KA has been associated with many short-term benefits, concerns remain about its long-term consequences. Some doubts have also been raised about the possible restriction on using KA based on the degree of preoperative varus, valgus, and flexion deformity [30]. In a recent study by Howell et al. [31], they observed that treating patients with KA-TKA without restrictions on preoperative deformity did not negatively affect the 10-year implant survival and level of function.

Assuming that KA has been associated with many short-term benefits, we wanted to evaluate and analyze short-term clinical, psychological, and functional outcomes in patients undergoing total knee replacement with KA, with the hypothesis of verifying a rapid recovery in the immediate postoperative period. Specifically, this study aims to compare the results of several specific clinical, psychological, and functional scores before surgery, the day after surgery, and on postoperative day 14.

## 2. Materials and Methods

The investigation was prospectively performed on a consecutive series of 12 patients undergoing total knee replacement surgery with kinematic alignment from May 2022 until July 2022. The inclusion criteria of the study are the following: patients who presented with severe knee osteoarthritis (Kellgren and Lawrence grade 3–4) [32] with pain and functional impotence; patients available for questionnaire administration and preoperative and postoperative evaluations (on postoperative day 1 and day 14). The exclusion criteria are patients undergoing revision knee replacement, patients undergoing knee replacement with techniques other than kinematic alignment, and patients who had hip replacements on the same limb. Patients participating in the study had to complete the same questionnaires before surgery, the day after surgery, and postoperative day 14. The questionnaires administered were

- Visual Analogue Scale (VAS) allows to estimate the intensity of pain experienced by the patient, which can be represented in a numerical scale from zero (corresponding to no pain) to ten (representing the highest level of pain);
- Short Form-12 (SF-12) is a questionnaire consisting of two sections, the first dealing with assessing physical aspects (PCS-Physical Component Summary, SF-12 PS) and the second assessing related aspects of mental health and well-being (MCS-Mental Component Summary, SF-12 MS);
- KSS allows assessment of the functional capabilities of the operated knee;
- Patient Health Questionnaire-9 (PHQ-9) is a scale for determining and monitoring the severity of depression;
- Knee Injury and Osteoarthritis Outcome Score-Physical Function Shortform (KOOS-PS) is a short questionnaire to assess the physical function of the knee.

For the conversion of questionnaires to score, the Orthotoolkit software (2023 version) was used for the VAS, SF-12 (PS and MS), PHQ-9, and KSS scales, while the conversion table proposed by Perruccio et al. was used for the KOOS-PS questionnaire score [33].

### 2.1. Rehabilitation Therapy

After surgery, patients received rehabilitation therapy. The main objectives of the rehabilitation protocol are represented by pain symptom control, joint mobility recovery, maintenance of good muscle tone-trophism, as well as the recovery of autonomy in walking and daily activities. Furthermore, another purpose of physical therapy treatment is to provide adequate preparation for the patient to know the fundamental steps in order to achieve a satisfactory recovery of mobility after the rehabilitation process at the hospital facility. The use of crutches is recommended. Specifically, during hospitalization, the patient is taught to walk with crutches and rehabilitated in stepping, changing direction, transferring from sitting to lying, and postural transitions from sitting to supine. Patients are also educated on the use of stairs before discharge. The exercises that are performed include isometric contractions of the quadriceps, concentric contractions of the quadriceps from sitting and supine position, straight leg raise, concentric contractions of the hip extensors, and concentric contractions of the hip abductors.

### 2.2. Statistical Analysis

The analysis was performed using SPSS software version 26 (IBM SPSS Statistics, Chicago, IL, USA). Parametric results were expressed as mean ± standard deviation (SD) and non-parametric distributions of the data are expressed as median (25th–75th percentile). The *t* test was the preferred method used to compare parametric, unmatched, and continuous variables. Wilcoxon matched-pairs signed-rank test was used to compare nonparametric, matched, and continuous variables. To verify normality, the Shapiro–Wilk test was utilized. Significance was set at *p* value < 0.05.

## 3. Results

There were 12 patients undergoing knee replacement with kinematic alignment technique from May 2022 to July 2022 who were evaluated and submitted to the questionnaires. In addition, the subjects included ten female and two male patients. Demographic data showed a mean BMI value of 30.4 (±3.4) Kg/m^2^ and mean age of 71.8 (±7.2) years.

**Figure 1:**
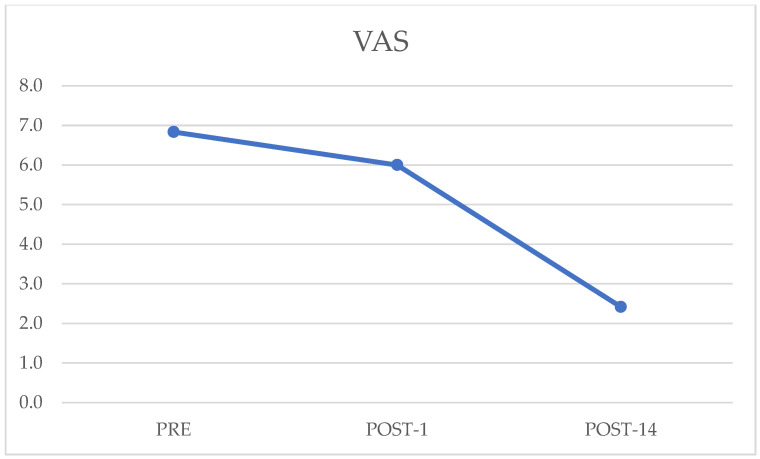
Preoperative, postoperative day 1, and postoperative day 14 values of Visual Analogue Scale (VAS) questionnaire.

The VAS test result in the preoperative evaluation was 6.8 ± 2.4. The mean value reported on the first postoperative day was 6 ± 2.3, while the value reported on the fourteenth postoperative day was 2.4 ± 0.9. Data analysis comparing preoperative and postoperative day 14 values showed statistically significant improvement (*p* = 0.0003); comparison of data from postoperative day 1 and postoperative day 14 also showed statistically significant improvement (*p* = 0.00004) (Figure 1).

**Figure 2:**
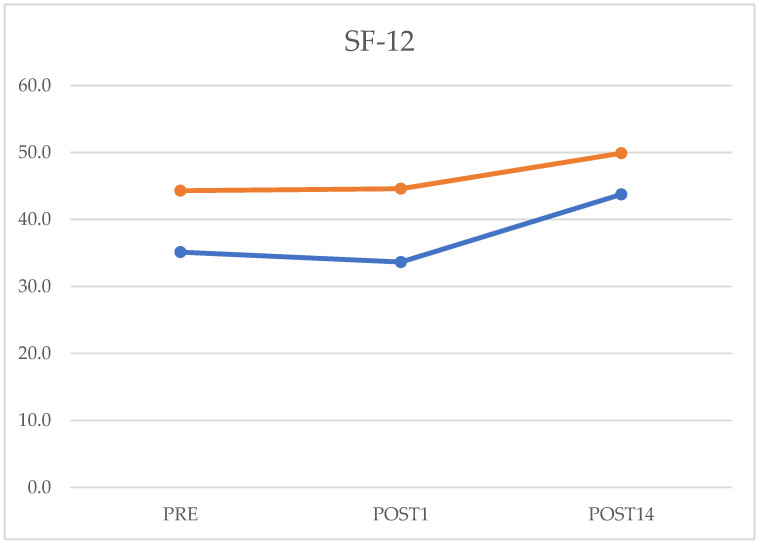
Preoperative, postoperative day 1, and postoperative day 14 values of Short Form-12 (SF-12) questionnaire. The orange line represents MCS-Mental Component Summary (SF-12 MS); the blue one represents PCS-Physical Component Summary (SF-12 PS).

The data obtained from the SF-12 PS questionnaire in the preoperative evaluation were 34 (6–33.75) on postoperative day one (4.25–33.75) and 44.5 on postoperative day 14 (5–45). After comparing the preoperative and postoperative data, it is possible to confirm that there was a statistically significant improvement (*p* = 0.0001), as well as in comparing the data on the first postoperative day and the fourteenth (*p* = 0.00001). Regarding the data obtained from the evaluation of the SF-12 MS questionnaire in the preoperative was 42.2 (4–42), the first postoperative day was 43.5 (3–47), and the fourteenth postoperative day was 50 (4–49.75). Comparing the preoperative and postoperative values on day 14 showed a statistically significant improvement (*p* = 0.002). There was a statistically significant improvement in studying the data of 1 and 14 days after the operation (*p* = 0.0007) (Figure 2).

**Figure 3:**
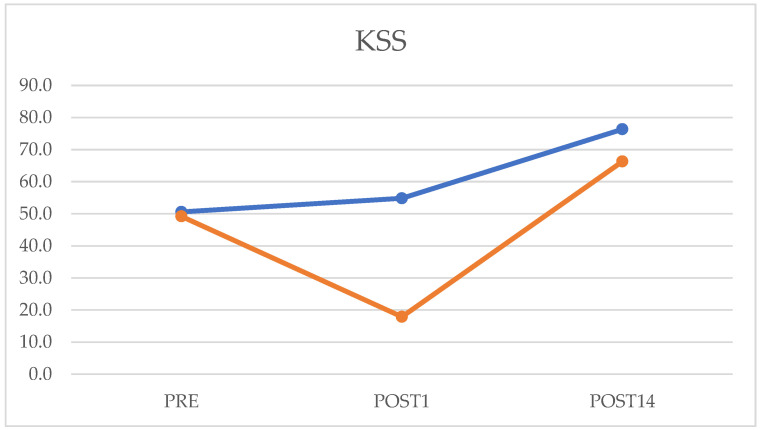
Preoperative, postoperative day 1, and postoperative day 14 values of Knee Society Score (KSS) questionnaire. The orange line represents the Knee Society Function Score (KSS-F); the blue one represents KSS.

The KSS result in preoperative was 50.6 ± 12.4, in postoperative was 54.8 ± 11.9, and on postoperative day 14 was 76.5 (75–79.75). After comparing the preoperative data with those of fourteen days after surgery, there was a statistically significant improvement (*p* = 0.0001), as well as in comparing the values of the first postoperative day with those of the fourteenth (*p* = 0.00003). The KSS-F resulted in the preoperative being 49.2 ± 6.3, the first postoperative was 15 (15–20), and on the fourteenth postoperative day was 66.2 ± 8.3. After analysis of the preoperative data with that of the fourteenth postoperative day, there was a statistically significant improvement (*p* = 0.0002). There was a statistically significant improvement in the analysis of the values1 and 14 days after the operation (Figure 3).

**Figure 4:**
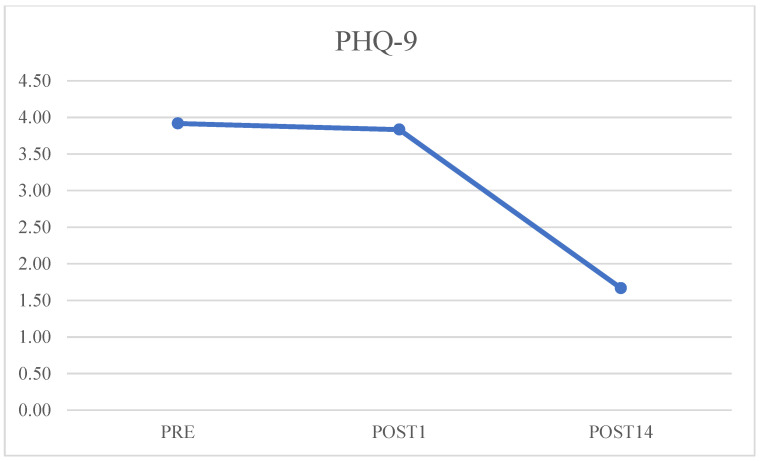
Preoperative, postoperative day 1, and postoperative day 14 values of Patient Health Questionnaire-9 (PHQ-9) questionnaire.

The PHQ-9 result after the preoperative questionnaire administration was four (2.25–4), on the first postoperative day it was three (3–4), and on day 14 it was one (0.25–2). After comparing preoperative and postoperative data on the 14th day, there was a statistically significant improvement (*p* = 0.0002). The same improvement is obtained by comparing data on the 1st day with the 14th day (*p* = 0.0003) (Figure 4).

**Figure 5:**
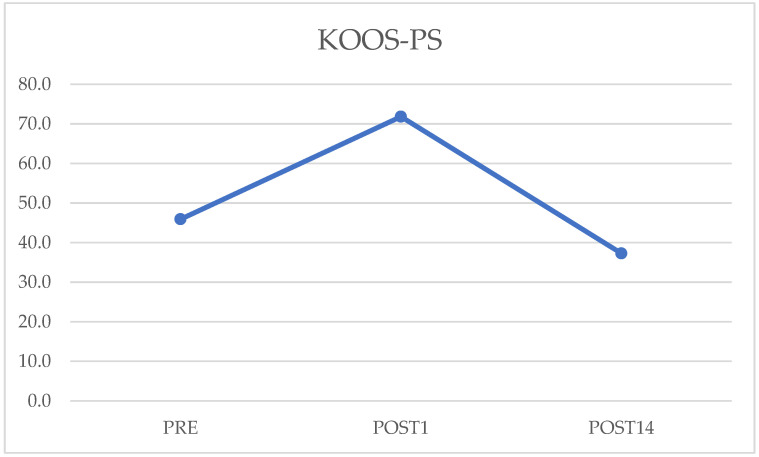
Preoperative, postoperative day 1, and postoperative day 14 values of Knee Injury and Osteoarthritis Outcome Score-Physical Function Shortform (KOOS-PS) questionnaire.

**Figure 6:**
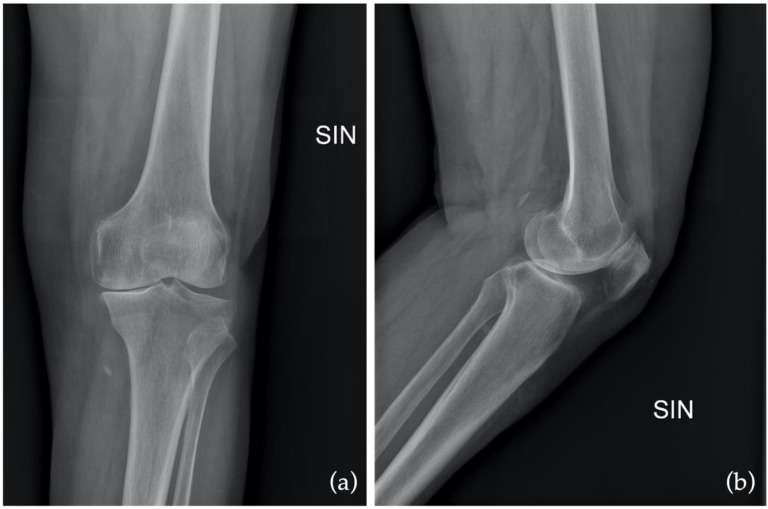
Preoperative X-ray in anteroposterior (AP) as (**a**) and in latero-lateral (LL) as (**b**).

**Figure 7:**
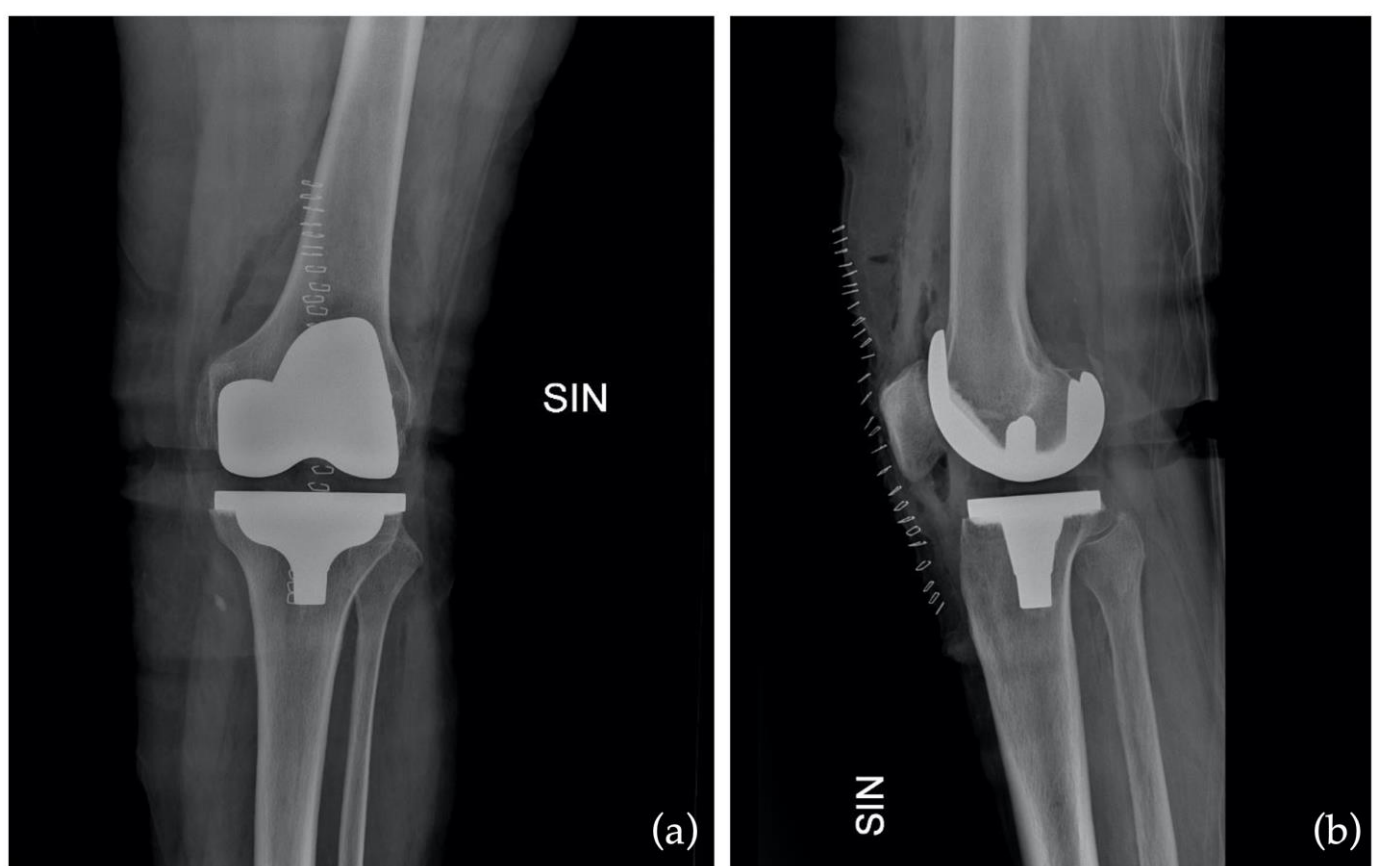
Postoperative X-ray in anteroposterior (AP) as (**a**) and in latero-lateral (LL) as (**b**).

Finally, the preoperative outcome of the KOOS-PS questionnaire is 46.25 (5–48), on the first postoperative day it is 71.8 (7.5–72.5), and on the fourteenth day it is 35.5 (6–35.5). Again, comparing preoperative data with those on postoperative day 14 showed improvement but was not statistically significant (*p* = 0.07). However, comparing those on the first postoperative and the fourteenth, there was a statistically significant increase (Figure 5). The values are summarized in Table 1. Examples of preoperative and postoperative images are shown in Figure 6 and Figure 7.

## 4. Discussion

This study reveals that patients who undergo knee prosthetic surgery using the kinematic alignment technique experience positive outcomes including clinical, functional, and psychological improvements, as early as two weeks after the procedure. This indicates that the technique promotes a faster recovery in various aspects important to patients’ lives. The results from multiple tests, including VAS, SF-12 PS, SF-12 MS, KSS, KSS-F, PHQ-9, and KOOS-PS, consistently demonstrated statistically significant improvements immediately after surgery and between the first and fourteenth days following the procedure.

These findings can be complemented by previous research, particularly the work of Bourne et al., which found that 11 to 19% of patients were dissatisfied with the final outcome of primary TKAs. The main predictors for dissatisfaction were unmet expectations, a low WOMAC score at one year of follow-up, preoperative pain at rest, and a postoperative complication requiring hospital readmission. As a result, it is essential for orthopedic surgeons to address patients’ expectations before TKA surgery to ensure they are realistic [20]. According to our results, KSS scores improved significantly right after surgery (*p* = 0.0001) and 14 days after the procedure (*p* = 0.00003), which is consistent with previous research indicating that KSS is better after kinematic alignment than mechanical alignment. Courtney et al.’s meta-analysis showed that while survivorship and complication rates were similar in both groups at short-term follow-up, the KA patient group had a higher combined postoperative KSS score than the group undergoing conventional treatment [34]. Jeremié et al.’s prospective study found that patients undergoing KA had better scores in both KSS and KOOS at the one-year follow-up, while Luo et al.’s meta-analysis found no significant difference in KOOS and KSS scores between KA and MA, with no increased complication rate [35,36]. Another older meta-analysis comparing scores in 229 patients with KA versus 229 patients with MA found that KSS function score did not show any significant differences, although a slight advantage was found for KSS pain in the KA group [37]. The proposed surgical technique by Howell et al. appears to be responsible for the positive functional and clinical outcomes observed in this study. This approach preserves the natural axis of the joint and respects soft tissues, particularly the ligamentous apparatus, without compromising postoperative correction [38]. An et al.’s retrospective study comparing 120 TKAs with MA and 90 with KM supports the claim that kinematic alignment leads to a less traumatic range of motion extension, reducing bone resection and soft tissue release. Additionally, there is no significant difference in the final overall limb alignment between the two techniques [39]. One common issue with TKA is the pain experienced by patients postoperatively, which can negatively impact rehabilitation and overall outcomes [40]. Our study demonstrates a significant reduction in pain symptoms as assessed by the VAS scale, starting from the first postoperative day and continuing through the fourteen postoperative days. This finding is consistent with the results of two systematic reviews that show the superiority of the KA-TKA technique in terms of pain relief [27,41]. In terms of knee function, patients showed a significant and rapid improvement in the first two weeks after surgery, with reasonable joint function and stability regained. Similar results were reported in a meta-analysis by Gao et al. that showed better functional outcomes for KA-TKA, with similar functions reported for both KA and MA at one-year follow-ups [9].

To date, only one prospective study has examined the initial results of different surgical techniques for total knee arthroplasty (TKA). The study compared kinematic alignment TKA (KA-TKA) with mechanical alignment TKA (MA-TKA) in two groups of 26 patients and collected clinical and functional scores before and after surgery at a mean follow-up of three months. The KA-TKA group showed statistically significant improvements in all scores tested, while the MA-TKA group demonstrated comparable results in the mental component score [42]. The survival rate of the KA-TKA implant has been shown to be comparable to that of the MA-TKA implant at short-term follow-up, according to a meta-analysis by Courtney et al. [34]. In a study by Howell et al., the first mid-term results of KA-TKA were reported, and they demonstrated a 10-year survival rate of more than 222 kinematically aligned TKA at 97.5% [31]. Future research could explore the impact of total knee replacement with kinematic alignment on adjacent joints. Proper knee alignment not only affects the knee joint itself, but also influences neighboring joints in a mutually dependent relationship [43]. For example, foot posture has been found to be related to the clinical and radiographic parameters of knee osteoarthritis. In Al-Bayati’s study, pain and functional status were evaluated in 150 patients with knee osteoarthritis, supination, neutrality, and pronation rates were also assessed. The results revealed that foot postural dysfunction should be considered during the evaluation and management of patients with knee osteoarthritis [44]. Ohi et al. performed a similar assessment by combining the anatomical axis of patients with knee osteoarthritis and three-dimensional foot posture. They analyzed 88 patients and found an association between the radiographic alignment of the knee in the frontal plane and the hallux valgus angle and the angle of the calcaneus to the floor in patients with medial knee osteoarthritis, particularly in varus-aligned knees. This association confirms the possible relationship between proper knee alignment and adjacent joints and may provide insights into the pathogenesis of altered foot posture in patients with knee osteoarthritis [45].

The present study has significant strengths. Firstly, it is one of the very few prospective studies in the literature that evaluates the use of kinematic alignment. Secondly, there is no other study that evaluates all the scores used in the assessment of our patients, providing for the first time such a comprehensive evaluation of the immediate postoperative period.

There are several limitations to our study that need to be acknowledged. Firstly, the sample size was small, and we only evaluated the clinical and functional outcomes of KA-TKA. Not having a control group is a significant limitation, and analyzing the same outcomes with other surgical techniques would be valuable in demonstrating the effectiveness of kinematic alignment. Therefore, prospective future studies are crucial to compare these results with mechanical alignment and validate our findings more robustly. Secondly, the follow-up period was limited, and a longer follow-up would be necessary to assess the longevity and durability of the KA-TKA technique. Nevertheless, we aimed to gather clinical and functional evidence in the immediate postoperative period that we observed in our patients. Due to the lack of data in the literature, we were unable to conduct a comprehensive comparison, which could have led to greater awareness of the best alignment technique for TKA. Therefore, the significance of our study is limited, but it serves as a reference for future studies in this area.

The results of this study confirm that KA provides patients with a rapid postoperative recovery and excellent clinical, psychological, and functional outcomes in a short period of time. These findings have significant clinical relevance and should be taken into account in the context of TKA and in the eventual choice of this surgical technique. Although this study requires further confirmation, we consider it significant as a starting point for future studies that may better overcome the limitations highlighted.

## 5. Conclusions

This study suggests that the kinematic alignment technique as a surgical treatment for KO allows the patient a fast postoperative recovery and better clinical, psychological, and functional results in a short time. Further studies are needed to confirm these results with a larger sample size and prospective randomized studies are essential to compare these results with mechanical alignment and validate our results more meaningfully.

## Figures and Tables

**Table 1:** Preoperative, postoperative day 1, and postoperative day 14 values of the questionnaires. Parametric results were expressed as mean ± standard deviation (SD), non-parametric distributions of the data are expressed as median (25th–75th percentile). * *p* < 0.05 (in the pre-post14 comparison). ^#^ *p* < 0.05 (in the post1–post14 comparison). Visual Analogue Scale (VAS), PCS-Physical Component Summary (SF-12 PS), MCS-Mental Component Summary (SF-12 MS), Knee Society Score (KSS), Knee Society Function Score (KSS-F), Patient Health Questionnaire-9 (PHQ-9), Knee Injury and Osteoarthritis Outcome Score-Physical Function Shortform (KOOS-PS).

	Preoperative	Post 1	Post 14
**VAS**	6.8 ± 2.4 (5–10)	6 ± 2.3 (2–10)	2.4 ±0.9 (1–4) *^#^
**SF-12 PS**	34 (6–33.75)	34 (4.25–33.75)	44.5 (5–45) *^#^
**SF-12 MS**	42.2 (4–42)	43.5 (3–47)	50 (4–49.75) *^#^
**KSS**	50.6 ± 12.4 (33–70)	54.8 ± 11.9 (38–74)	76.5 (75–79.75) *^#^
**KSS-F**	49.2 ± 6.3 (50–60)	15 (15–20)	66.2 ± 8.3 (55–80) *^#^
**PHQ-9**	4 (2.25–4)	3 (3–4)	1 (0.25–2) *^#^
**KOOS-PS**	46.25 (5–48)	71.8 (7.5–72.5)	35.5 (6–35.5) ^#^

## Data Availability

Data supporting the reported results can be found in the database generated during the study.

## References

[1] Anzillotti G., Conte P., Di Matteo B., Bertolino E.M., Marcacci M., Kon E. (2022). Injection of biologic agents for treating severe knee osteoarthritis: Is there a chance for a good outcome? A systematic review of clinical evidence. Eur. Rev. Med. Pharmacol. Sci..

[2] Giorgino R., Albano D., Fusco S., Peretti G.M., Mangiavini L., Messina C. (2023). Knee Osteoarthritis: Epidemiology, Pathogenesis, and Mesenchymal Stem Cells: What Else Is New? An Update. Int. J. Mol. Sci..

[3] Dell’isola A., Allan R., Smith S.L., Marreiros S.S.P., Steultjens M. (2016). Identification of clinical phenotypes in knee osteoarthritis: A systematic review of the literature. BMC Musculoskelet. Disord..

[4] Heidari B. (2011). Knee osteoarthritis prevalence, risk factors, pathogenesis and features: Part I. Casp. J. Intern. Med..

[5] Wallace I.J., Worthington S., Felson D.T., Jurmain R.D., Wren K.T., Maijanen H., Woods R.J., Lieberman D.E. (2017). Knee osteoarthritis has doubled in prevalence since the mid-20th century. Proc. Natl. Acad. Sci. USA.

[6] Hussain S.M., Neilly D.W., Baliga S., Patil S., Meek R. (2016). Knee osteoarthritis: A review of management options. Scott. Med. J..

[7] Mora J.C., Przkora R., Cruz-Almeida Y. (2018). Knee osteoarthritis: Pathophysiology and current treatment modalities. J. Pain Res..

[8] Filippo M., Laura M., Riccardo G., Valeria V., Eschweiler J., Maffulli N. (2022). Mesenchymal stem cells augmentation for surgical procedures in patients with symptomatic chondral defects of the knee: A systematic review. J. Orthop. Surg. Res..

[9] Gao Z., Long N., Zhang S., Yu W., Dai Y., Xiao C. (2020). Comparison of Kinematic Alignment and Mechanical Alignment in Total Knee Arthroplasty: A Meta-analysis of Randomized Controlled Clinical Trials. Orthop. Surg..

[10] Gunaratne R., Pratt D.N., Banda J., Fick D.P., Khan R.J., Robertson B.W. (2017). Patient Dissatisfaction Following Total Knee Arthroplasty: A Systematic Review of the Literature. J. Arthroplast..

[11] Varacallo M., Luo T.D., Johanson N.A. (2023). Total Knee Arthroplasty Techniques.

[12] Kurtz S., Mowat F., Ong K., Chan N., Lau E., Halpern M. (2005). Prevalence of Primary and Revision Total Hip and Knee Arthroplasty in the United States From 1990 Through 2002. J. Bone Jt. Surg. Am..

[13] Weber P., Gollwitzer H. (2021). Kinematic alignment in total knee arthroplasty. Oper. Orthop. Traumatol..

[14] Bellemans J., Colyn W., Vandenneucker H., Victor J. (2012). The Chitranjan Ranawat Award: Is Neutral Mechanical Alignment Normal for All Patients?: The Concept of Constitutional Varus. Clin. Orthop. Relat. Res..

[15] Ma C.B., Lee K.-Y., Schrumpf M.A., Majumdar S. (2005). Analysis of three-dimensional in vivo knee kinematics using dynamic magnetic resonance imaging. Oper. Tech. Orthop..

[16] Eckhoff D.G., Bach J.M., Spitzer V.M., Reinig K.D., Bagur M.M., Baldini T.H., Flannery N.M. (2005). Three-Dimensional Mechanics, Kinematics, and Morphology of the Knee Viewed in Virtual Reality. J. Bone Jt. Surg..

[17] Coughlin K.M., Incavo S.J., Churchill D.L., Beynnon B.D. (2003). Tibial axis and patellar position relative to the femoral epicondylar axis during squatting. J. Arthroplast..

[18] Iranpour F., Merican A.M., Dandachli W., Amis A.A., Cobb J.P. (2010). The Geometry of the Trochlear Groove. Clin. Orthop. Relat. Res..

[19] Mannan A., Saber A.Y., Waterson B., Roberton A., Toms A. (2022). Mechanical Alignment in Total Knee Arthroplasty for Varus Knee Osteoarthritis Leads to Significant Tibial Bone Loss. Cureus.

[20] Bourne R.B., Chesworth B.M., Davis A.M., Mahomed N.N., Charron K.D.J. (2010). Patient Satisfaction after Total Knee Arthroplasty: Who is Satisfied and Who is Not?. Clin. Orthop. Relat. Res..

[21] Howell S.M., Kuznik K., Hull M.L., Siston R.A. (2008). Results of an Initial Experience with Custom-fit Positioning Total Knee Arthroplasty in a Series of 48 Patients. Orthopedics.

[22] Gu Y., Howell S.M., Hull M.L. (2017). Simulation of total knee arthroplasty in 5° or 7° valgus: A study of gap imbalances and changes in limb and knee alignments from native. J. Orthop. Res. Off. Publ. Orthop. Res. Soc..

[23] Nam D., Lin K.M., Howell S.M., Hull M.L. (2014). Femoral bone and cartilage wear is predictable at 0° and 90° in the osteoarthritic knee treated with total knee arthroplasty. Knee Surg. Sports Traumatol. Arthrosc. Off. J. ESSKA.

[24] Nedopil A.J., Singh A.K., Howell S.M., Hull M.L. (2018). Does Calipered Kinematically Aligned TKA Restore Native Left to Right Symmetry of the Lower Limb and Improve Function?. J. Arthroplast..

[25] Ji H.-M., Han J., Jin D.S., Seo H., Won Y.-Y. (2016). Kinematically aligned TKA can align knee joint line to horizontal. Knee Surg. Sports Traumatol. Arthrosc. Off. J. ESSKA.

[26] Niki Y., Nagura T., Nagai K., Kobayashi S., Harato K. (2018). Kinematically aligned total knee arthroplasty reduces knee adduction moment more than mechanically aligned total knee arthroplasty. Knee Surg. Sports Traumatol. Arthrosc. Off. J. ESSKA.

[27] Yoon J.-R., Han S.-B., Jee M.-K., Shin Y.-S. (2017). Comparison of kinematic and mechanical alignment techniques in primary total knee arthroplasty: A meta-analysis. Medicine.

[28] Giustra F., Bosco F., Cacciola G., Risitano S., Capella M., Bistolfi A., Massè A., Sabatini L. (2022). No Significant Differences in Clinical and Radiographic Outcomes between PCL Retained or Sacrificed Kinematic Aligned Medial Pivot Total Knee Arthroplasty in Varus Knee. J. Clin. Med..

[29] Xu J., Cao J.Y., Luong J.K., Negus J.J. (2019). Kinematic versus mechanical alignment for primary total knee replacement: A systematic review and meta-analysis. J. Orthop..

[30] Almaawi A.M., Hutt J.R., Masse V., Lavigne M., Vendittoli P.-A. (2017). The Impact of Mechanical and Restricted Kinematic Alignment on Knee Anatomy in Total Knee Arthroplasty. J. Arthroplast..

[31] Howell S.M., Shelton T.J., Hull M.L. (2018). Implant Survival and Function Ten Years After Kinematically Aligned Total Knee Arthroplasty. J. Arthroplast..

[32] Kohn M.D., Sassoon A.A., Fernando N.D. (2016). Classifications in Brief: Kellgren-Lawrence Classification of Osteoarthritis. Clin. Orthop. Relat. Res..

[33] Perruccio A., Lohmander L.S., Canizares M., Tennant A., Hawker G., Conaghan P., Roos E., Jordan J., Maillefert J.-F., Dougados M. (2008). The development of a short measure of physical function for knee OA KOOS-Physical Function Shortform (KOOS-PS)—An OARSI/OMERACT initiative. Osteoarthr. Cartil..

[34] Courtney P.M., Lee G.-C. (2017). Early Outcomes of Kinematic Alignment in Primary Total Knee Arthroplasty: A Meta-Analysis of the Literature. J. Arthroplast..

[35] Jeremić D.V., Massouh W.M., Sivaloganathan S., Rosali A.R., Haaker R.G., Rivière C. (2020). Short-term follow-up of kinematically vs. mechanically aligned total knee arthroplasty with medial pivot components: A case-control study. Orthop. Traumatol. Surg. Res. OTSR.

[36] Luo Z., Zhou K., Peng L., Shang Q., Pei F., Zhou Z. (2020). Similar results with kinematic and mechanical alignment applied in total knee arthroplasty. Knee Surg. Sports Traumatol. Arthrosc. Off. J. ESSKA.

[37] Woon J.T.K., Zeng I.S.L., Calliess T., Windhagen H., Ettinger M., Waterson H.B., Toms A.D., Young S.W. (2018). Outcome of kinematic alignment using patient-specific instrumentation versus mechanical alignment in TKA: A meta-analysis and subgroup analysis of randomised trials. Arch. Orthop. Trauma Surg..

[38] Howell S.M. (2019). Calipered Kinematically Aligned Total Knee Arthroplasty: An Accurate Technique That Improves Patient Outcomes and Implant Survival. Orthopedics.

[39] An V.V., Twiggs J., Leie M., Fritsch B.A. (2019). Kinematic alignment is bone and soft tissue preserving compared to mechanical alignment in total knee arthroplasty. Knee.

[40] Li J., Ma Y., Xiao L. (2019). Postoperative Pain Management in Total Knee Arthroplasty. Orthop. Surg..

[41] Li Y., Wang Y., Yang M., Wang S. (2018). Does Kinematic Alignment Improve Short-Term Functional Outcomes after Total Knee Arthroplasty Compared with Mechanical Alignment? A Systematic Review and Meta-analysis. J. Knee Surg..

[42] Luceri F., Sosio C., Sirtori P., Battistella D., Zuffada M., Ulivi M., Meroni V., Marmotti A., Mangiavini L., Peretti G.M. (2020). Kinematic versus mechanical alignment in total knee arthroplasty: A preliminary study. J. Biol. Regul. Homeost. Agents..

[43] Akaltun M.S., Koçyiğit B.F. (2021). Assessment of foot posture and related factors in patients with knee osteoarthritis. Arch. Rheumatol..

[44] Al-Bayati Z., Benlidayi I.C., Gokcen N. (2018). Posture of the foot: Don’t keep it out of sight, out of mind in knee osteoarthritis. Gait Posture.

[45] Ohi H., Iijima H., Aoyama T., Kaneda E., Ohi K., Abe K. (2017). Association of frontal plane knee alignment with foot posture in patients with medial knee osteoarthritis. BMC Musculoskelet. Disord..

